# Parotitis as an Initial Symptom of Kawasaki Disease

**DOI:** 10.1155/2017/5937276

**Published:** 2017-05-11

**Authors:** Koji Yokoyama

**Affiliations:** Department of Pediatrics, Japanese Red Cross Wakayama Medical Center, Wakayama, Japan

## Abstract

We report the case of a 13-month-old boy who developed right side parotitis as a first symptom of Kawasaki disease (KD). The data presented herein suggest that physicians should be aware that nonsuppurative parotitis is a possible manifestation of KD.

## 1. Case Presentation

**Figure 1:**
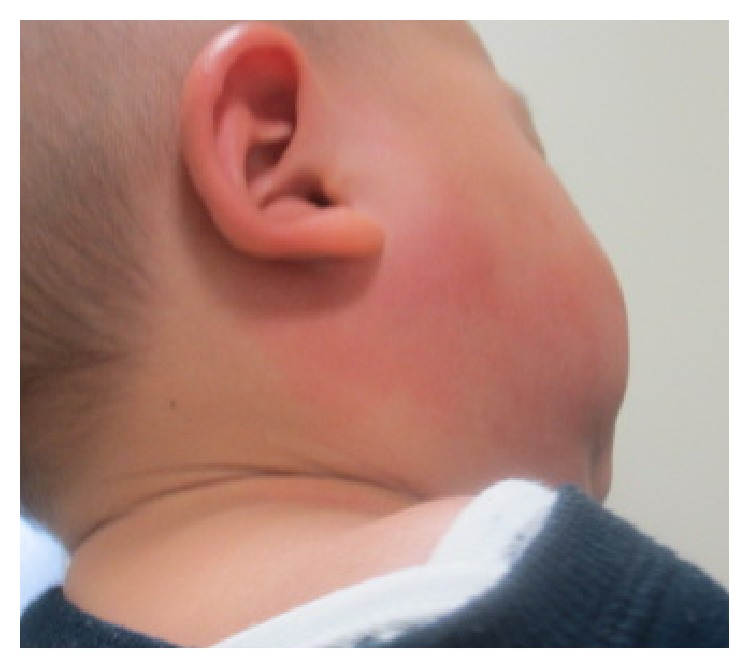
Photograph showing a reddish mass on the right jaw.

**Figure 2:**
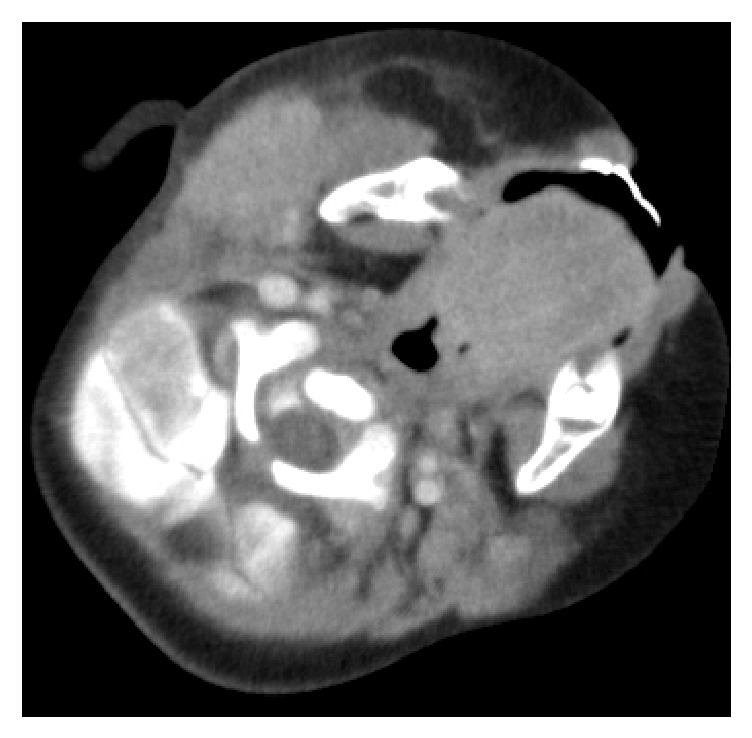
An enhanced computed tomography scan of the pharynx showed increased intensity in the right parotid grand.

**Figure 3:**
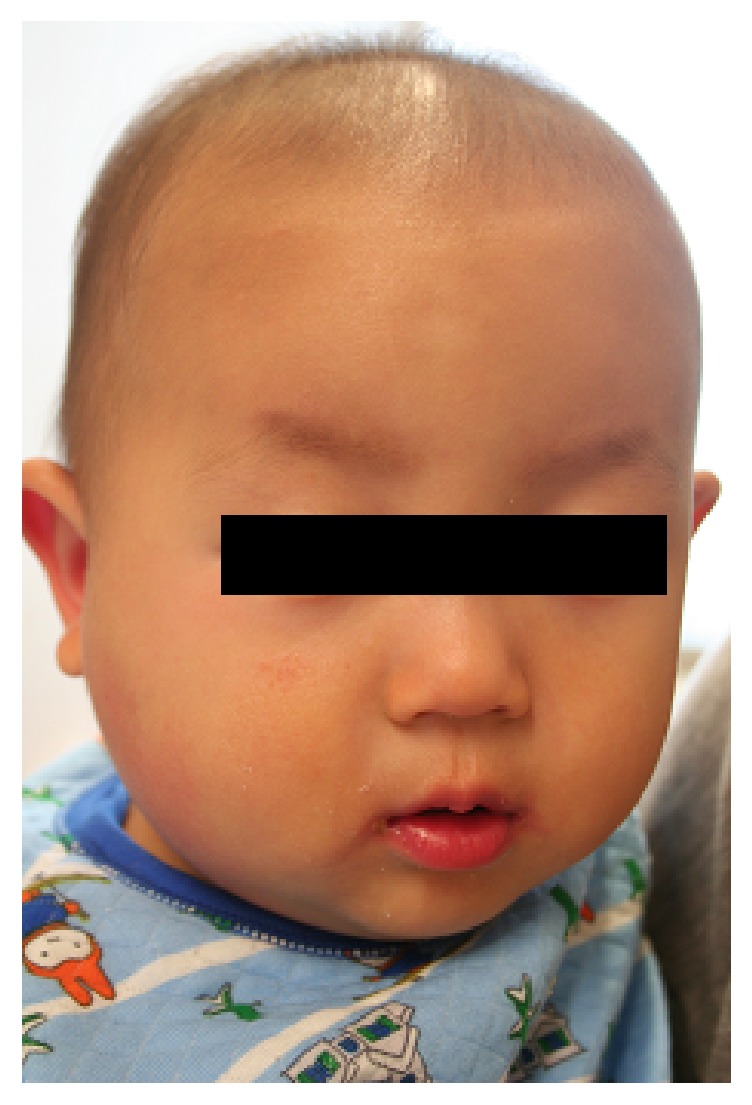
Photo showing lip redness and conjunctival injection.

A 13-month-old boy was admitted to our hospital with a 3-day history of fever and right side enlarged parotid gland. There was no recent history of travel, contact with sick individuals, or exposure to pets. He had not been vaccinated against mumps. Physical examination revealed a body temperature of 39.5°C and a respiratory rate of 48 breaths per minute. A painful, erythematous, warm hard mass measuring 2 × 4 cm was identified between the area posterior to the right ear and the area inferior to the jaw (Figure 1). Laboratory findings on admission were as follows: leukocyte count, 28200/mm3 (67% neutrophils, 26% lymphocytes, and 1% atypical lymphocytes), C-reactive protein, 7.73 mg/dl, and amylase, 362 IU/l (normal value, 40–120). Other laboratory findings (pancreatic type amylase, antinuclear antibodies, anti-Sjögren's syndrome-related antigen A/B antibodies, rheumatoid factor, and gamma globulin level) were normal. The patient was negative for antimumps virus immunoglobulin G and M. Blood culture was also negative. Real time-polymerase chain reaction was negative for mumps genome in urine or blood. An enhanced computed tomography scan of the pharynx revealed increased intensity in the right parotid grand, and the adjacent lymph nodes were enlarged (Figure 2). The patient received ampicillin/sulbactam and clindamycin for 2 days to treat presumed suppurative parotitis. On the 5th day of illness, he developed lip redness, conjunctival injection, reddening of the BCG injection site, and edema of the hands and feet (Figure 3). He was then given a diagnosis of Kawasaki disease (KD); he became afebrile 22 hours after administration of intravenous gamma globulin (2 g/kg) and aspirin. No coronary artery involvement was detected by echocardiography, either on admission or 4 weeks later.

## 2. Discussion

The above data provide potentially important clinical information, for example, that parotitis may present as the first symptom of KD. KD is the most common cause of multisystem vasculitis in children. The clinical and epidemiological features of KD indicate a probable infectious cause [1]. It has been hypothesized that infectious agents associated with KD trigger a complex and incompletely understood cascade of inflammation in susceptible children [2, 3]. KD is a type of systemic vasculitis, although here we focused on cervical symptoms. Most (65%) patients showing otolaryngologic manifestations of KD present with cervical lymphadenopathy [4]. Three previous reports describe parotitis associated with KD [5–7]. No study has reported an association between KD and other types of sialoadenitis, including submandibular or sublingual sialoadenitis. This anatomical anomaly with respect to the inflammatory site may provide information relevant to identifying the mechanism underlying the pathology of KD.

In conclusion, physicians should be aware that nonsuppurative parotitis may be a manifestation of KD.
